# New Mobile Device to Measure Verticality Perception: Results in Young Subjects with Headaches

**DOI:** 10.3390/diagnostics10100796

**Published:** 2020-10-07

**Authors:** Daniel Rodríguez-Almagro, Esteban Obrero-Gaitán, Rafael Lomas-Vega, Noelia Zagalaz-Anula, María Catalina Osuna-Pérez, Alexander Achalandabaso-Ochoa

**Affiliations:** Department of Health Science, University of Jaén, Paraje Las Lagunillas s/n, 23071 Jaén, Spain; dra00005@red.ujaen.es (D.R.-A.); eobrero@ujaen.es (E.O.-G.); nzagalaz@ujaen.es (N.Z.-A.); mcosuna@ujaen.es (M.C.O.-P.); aaochoa@ujaen.es (A.A.-O)

**Keywords:** mobile applications, diagnostic equipment, headache, migraine, postural balance, visual motor coordination, vestibular function tests

## Abstract

The subjective visual vertical (SVV) test has been frequently used to measure vestibular contribution to the perception of verticality. Recently, mobile devices have been used to efficiently perform this measurement. The aim of this study was to analyze the perception of verticality in subjects with migraines and headaches. A cross-sectional study was conducted that included 28 patients with migraine, 74 with tension-type headache (TTH), and 93 healthy subjects. The SVV test was used through a new virtual reality system. The mean absolute error (MAE) of degrees deviation was also measured to qualify subjects as positive when it was greater than 2.5°. No differences in the prevalence of misperception in verticality was found among healthy subjects (31.18%), migraineurs (21.43%), or those with TTH (33.78%) (*p* = 0.480). The MAE was not significantly different between the three groups (migraine = 1.36°, TTH = 1.61°, and healthy = 1.68°) (F = 1.097, *p* = 0.336, and η2 = 0.011). The perception of verticality could not be explained by any variable usually related to headaches. No significant differences exist in the vestibular contribution to the perception of verticality between patients with headaches and healthy subjects. New tests measuring visual and somatosensory contribution should be used to analyze the link between the perception of verticality and headaches.

## 1. Introduction

Assessment of the perception of verticality is increasingly used in patients with disorders of upright body orientation [1]. It is based on a gravitational input processed in the central nervous system (CNS) from vestibular, visual, and somatosensory information [2,3] that can be measured through the use of touch, which is called haptic vertical, or by estimating the position of one’s own body without the help of visual inputs, which is called subjective postural vertical (SPV) [4]. However, visual estimation of the vertical (i.e., visual vertical (VV)) is the most common test used to assess the perception of verticality in research and clinical practice [5]. The subjective visual vertical (SVV) test consists of adjusting a random-oriented line to the vertical position without the help of visual references. The initial orientation is usually between 30° and 60° right or left. The consensual values considered normal for SVV are between −2.5° and 2.5° with respect to the actual vertical [6]. 

It is believed that SVV tests estimate the ability of a person to perceive the gravitational vertical, and a tilt in SVV indicates vestibular imbalance in the roll plane and, thus, injuries to the utricle or its connecting nerves [7]. Although measurements of the perceived visual vertical disclose mainly vestibular dysfunctions when no cues to visual spatial orientation are provided during testing [8], several studies have found that the SVV is altered in neurological patients, mainly with stroke [9]; in subjects with spinal diseases [10]; and in patients with peripheral vestibular disorders [11,12].

An SVV test is performed classically using the bucket method, which is an easily performed and reliable bedside test for determining monocular and binocular SVV that costs less than $5 [13]. However, the bucket test is a limited method that does not allow automated data storage and is not sufficiently versatile to be able to implement different versions of VV measurements. For this reason, in recent years, various wearable methods have been created using virtual reality and mobile devices [7,14]. Most of these new methods have been tested in healthy subjects to analyze the methods′ feasibility and reliability. However, experiences with subjects with health problems are scarce.

Vestibular, visual, and somatosensory systems play a major role in verticality perception [2,3]. Furthermore, it is frequently observed how disfunction in these three systems appear in conjunction with headache, taking an important part in headache development [15,16,17]. The possibility that patients with headaches could present some alterations in any of these three systems that may induce a misperception of VV has turned headache disorders into a study issue in relation to alteration of VV.

Headaches are a significant public health problem that affect approximately 40.5% of the global population, taking into account both migraines and tension-type headaches (TTHs) [18]. This problem is more frequent among females, university students, and urban residents [19]. Several studies have looked for an alteration of verticality in subjects with migraines and TTHs with contradictory results; some studies found no significant differences between subjects with primary headache disorders (PHD) and healthy subjects [20,21,22], while others found differences between healthy subjects and subjects who suffered PHD [23,24]. In view of these results, it is of interest to assess the differences in perception of VV between patients with migraine and TTH, and healthy subjects. 

Additionally, some works have shown possible common factors between headache and verticality perception. A recent study of Martins et al. [25] showed a relationship between sleep disturbances and modifications in perception of verticality. Furthermore, it has been possible to observe the influence of physical activity in verticality perception [26]. In the same way, both sleep disturbances [27,28] and physical activity [29] also related to the presence of headaches and migraines. In view of the above, it might be asked whether these factors are able to explain the presence or magnitude of the alteration in perception of verticality in patients with headaches.

This study is a feasibility analysis of a new device for measuring SVV, previously validated in healthy people. The main objective of this work was to analyze the possible differences in visual perception of verticality between subjects with migraines, subjects with TTHs, and healthy subjects using a new mobile device. The secondary objective was to identify which variables usually associated with headaches could be related to SVV deviation in young students.

## 2. Materials and Methods 

### 2.1. Study

To meet the objectives of this work, a cross-sectional observational study was designed, developed in accordance with the guidelines for the communication of observational studies established in the Strengthening the Reporting of Observational studies in Epidemiology (STROBE) Statement [30]. This study was carried out in accordance with the Helsinki declaration, good clinical practice, and all applicable laws and regulations and was approved by the Ethics Committee of the University of Jaén (reference number ABR. 17./7.TFM). All participants signed informed consent document.

For this study, the participants contacted us as response to posters and digital advertisements published at the University of Jaén (Jaén, Spain). The data were collected between the months of October and December 2018 at the University of Jaén. The participants had to be young subjects, university students, and older than 18 years who did not suffer from cognitive disorders; eye diseases; previous head or neck trauma; any type of acquired brain damage (ischemic or hemorrhagic stroke or damage resulting from intracranial intervention); any systemic disease with visual, vestibular, central, or musculoskeletal involvement; neuromuscular disease; or presence of neoplasia at the visual, vestibular, or central level.

### 2.2. Sample Size Calculation

The sample size calculation was carried out using the data obtained in the study of Asai et al. [23]. Taking into account a prevalence of migraines of approximately 20% [18] and a total prevalence of headaches of approximately 52% [31], to obtain between-group significant differences with an alpha error of 5% and a power of 80%, a minimum of 179 subjects was required. 

### 2.3. Subjects

**Figure 1:**
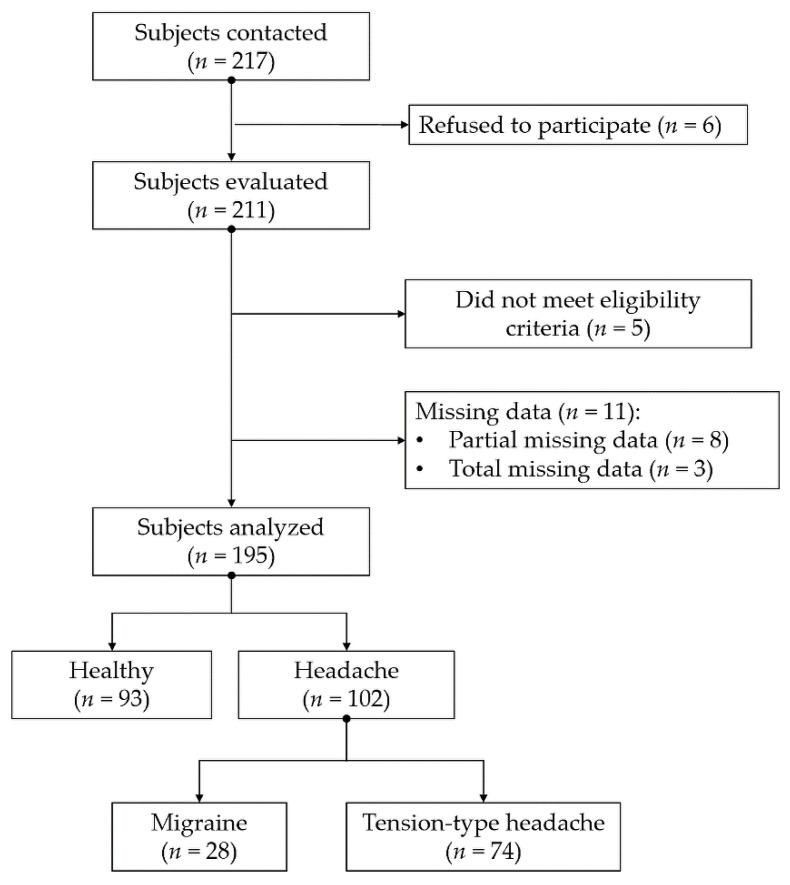
Flow chart of the participant selection process.

Two hundred and seventeen subjects were initially contacted during the month of September 2018. Of these 217 subjects, 211 were selected to participate in the present investigation after having been duly informed. Finally, 195 subjects completed all of the questionnaires and evaluations planned in the study. The selection process is graphically represented in Figure 1. 

All subjects were evaluated by a physician (F.H.) who verified compliance with the eligibility criteria as well as compliance with the criteria described in the third edition of “The International Classification of Headache Disorders” [32].

### 2.4. Measurements

**Figure 2:**
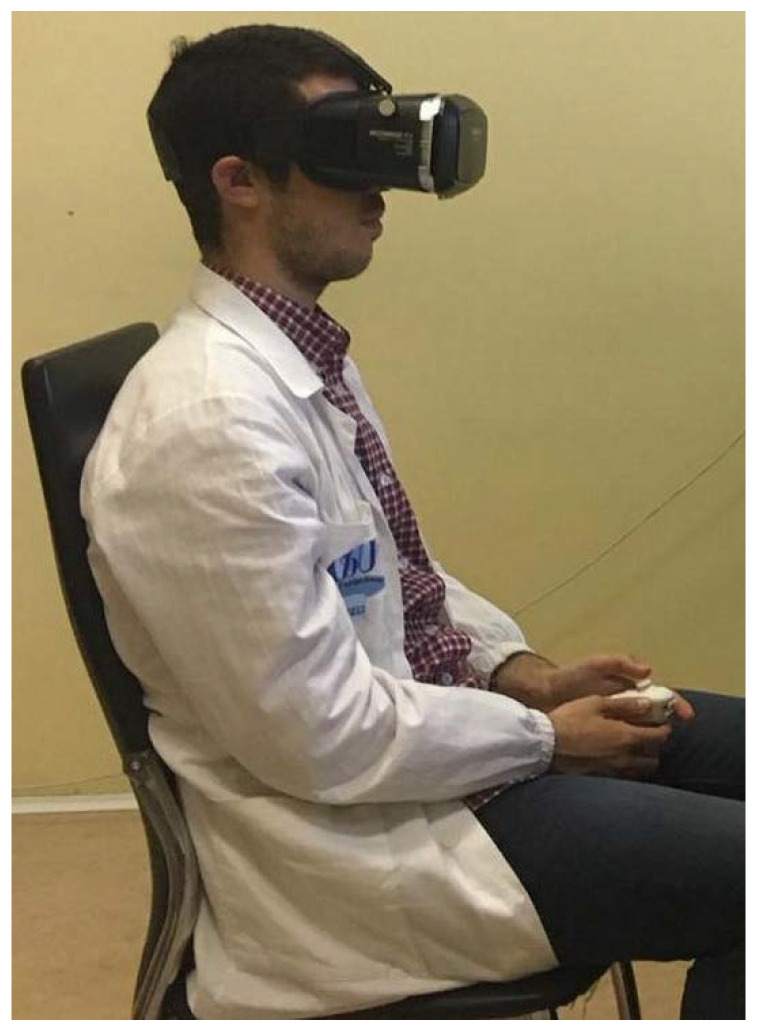
Participant using the mobile device to measure subjective visual vertical (SVV).

First, sociodemographic variables were recorded, including gender, age, height, weight, years at university, smoking habit, and physical activity. To quantify deviation of the perceived vertical from the theoretical vertical, the static SVV test was used through a new virtual reality system [14] during the interictal phase of headache process. The virtual reality system requires a mobile device placed into the back of a headset and a Google Cardboard-enabled application (Sistema de Realidad Virtual para Detección y Tratamiento de las Patologías Posturales y del Equilibrio, University of Jaén, Jaén, Spain, 2019) to generate a pair of stereo images (Figure 2). The test was performed in a quiet environment with dim lighting while the subject sat comfortably with their back straight and their feet uncrossed and resting on the ground. When the subject was ready, the evaluator started the test from the web application. Firstly, the virtual reality system set the line in a random position between 30° and 60° right or left. Then, the subject rotated the line using the joystick until they perceived that it was close to vertical and, then, confirmed the result with an action button. The subject had 30 second to perform each test. To calculate SVV, six measurements were made, from which the mean deviation of the perceived vertical with respect to the theoretical vertical was obtained. The mean absolute error (MAE) was calculated as the average value of the error made in each attempt, without taking the direction of deviation into account. For treatment of the dependent variable in this study, deviation of the SVV value from normal was also considered, taking as normal values those between −2.5° (left deviation) and 2.5° (right deviation) [5]. The device was validated and showed good reliability (Intraclass Correlation Coefficient (ICC) = 0.85; 95% confidence interval (CI) = 0.75–0.92) [14]. The evaluation was always made with visual correction if the patient had it prescribed.

Headache-related disability as well as its frequency and intensity were assessed using the Spanish version of the migraine disability assessment (MIDAS) questionnaire [33]. This instrument is made up of seven items, the first five of which focus on three dimensions of daily life that can be affected by headaches while the remaining two items refer to the frequency and intensity of the headache. The sum of the scores of the first five items provides the degree of disability related to the headache, while the sixth and seventh items indicate the frequency and intensity of the headache, respectively. The Spanish version of the questionnaire has good reliability and validity properties [33].

The disability associated with neck pain was evaluated with the Spanish version of the “Neck Disability Index” (NDI) questionnaire [34], which is a self-administered questionnaire with 10 sections. Each of the sections offers six possible answers that represent six progressive levels of functional capacity that are scored from 0 to 5. The reliability values of this questionnaire are very high (ICC = 0.989), and it also has good internal consistency (Cronbach’s α = 0.913) [34].

Sleep quality was also included as a predictor variable, due to the relationship that has been reported between the perception of verticality and the variables related to sleep [35]. To measure sleep quality, the Spanish version of the “Medical Outcomes Study Sleep Scale” (MOS-SS) was used [36], which is a self-administered questionnaire composed of 12 items, from which six subscales are extracted. From the MOS-SS questionnaire, the variables used were sleep disturbances (ICC = 0.78; 95% CI = 0.62–0.88), daytime sleepiness (ICC = 0.57; 95% CI = 0.30–0.75), sleep adequacy (ICC = 0.75; 95% CI = 0.56–0.87), snoring (ICC = 0.84; 95% CI = 0.71–0.91), waking up briefly at night due to respiratory reasons or headache (ICC = 0.84; 95% CI = 0.71–0.91), and optimal sleep (ICC = 0.76; 95% CI = 0.58–0.87), for which the reliability values were between moderate and high [36].

### 2.5. Statistical Analysis

Data management and analysis was carried out using the SPSS statistical package, version 23.0 (SPSS Inc, Chicago, IL, USA). The level of statistical significance was established as *p* < 0.05. The data were described using means and standard deviations for continuous variables and using frequencies and percentages for categorical variables. To determine the normality of continuous variables, the Kolmogorov–Smirnov test was used, while the Levene’s test of equality of variances was used to determine the homoscedasticity of the samples.

To analyze the differences in the perception of verticality with respect to the theoretical vertical between healthy subjects, subjects with TTHs, or those with migraines, one-way analysis of variance (ANOVA) was used, while eta-squared (η2) was used to express the effect size. To evaluate differences in the prevalence of SVV alterations (SVV more than 2.5° of deviation) between subjects with TTHs or migraines and healthy subjects, the chi-square test was used.

Given the binary nature of the “alteration in the perception of verticality” variable (MAE > 2.5 or not), univariate logistic regression was used to identify which variables are related to it. The independent variables comprised sociodemographic variables; frequency, intensity, and disability associated with headaches; disability associated with neck pain; and variables related to sleep.

To identify the variables related to the degree of deviation of the perceived vertical from the theoretical vertical, univariate linear regression was used, given the continuous nature of the dependent variable. The independent variables for this analysis were the same as those used in the logistic regression.

## 3. Results

Of the total number of participants who completed the study, 111 were women and 84 were men. Twenty-eight subjects met the criteria for migraines, 74 met the criteria for TTHs, and 93 subjects were classified as healthy. The total prevalence of headaches in the present study was 52.3%, with 72.5% TTH and 27.5% migraines. The prevalence of verticality alterations was very similar between the three groups (Table 1). There were no statistically significant differences in the prevalence of SVV alteration (*p* = 0.480).

**Figure 3:**
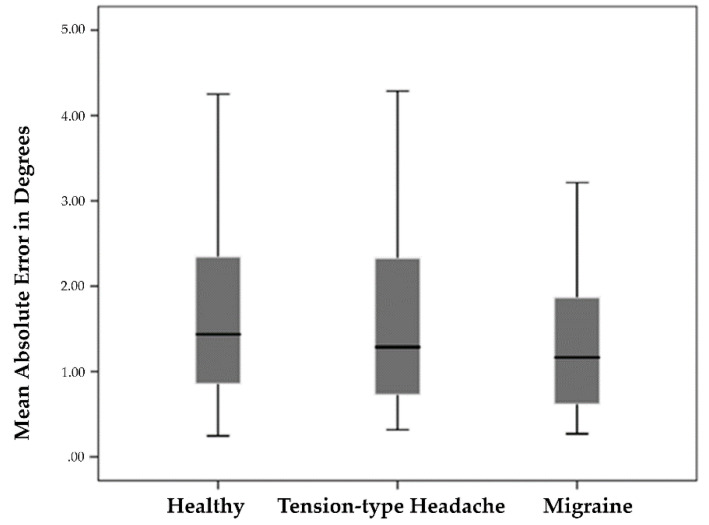
Between-group differences in mean absolute error in estimating subjective visual vertical (SVV).

One-way ANOVA showed no statistically significant between-group differences in the MAE between subjects with migraines or TTHs and healthy subjects (F = 1.097, *p* = 0.336, and η2 = 0.011). The results are graphically shown in Figure 3.

The logistic regression performed to identify the variables related to alterations in the perception of verticality (Table 2) and the linear regression used to establish the variables that explained the degree of deviation in the MAE (Table 3) did not show statistically significant associations. An association was only found at the limit of statistical significance (*p* = 0.054) between headache-related disability and the degree of MAE deviation.

## 4. Discussion

This work aimed to analyze the differences in the visual perception of verticality between healthy subjects, migraineurs, and those with TTHs using a new mobile device in conjunction with virtual reality glasses. The test was carried out without any great difficulties, and the results were stored within the developed application. The duration of each test was no more than 5 min, including the placement of the device, familiarization with it, and the performance of all required attempts. 

During the development of this research, it was observed that contribution of the vestibular system to the perception of verticality remained stable in young students who present this pathology. No differences were found in the perception of verticality between healthy subjects, subjects with TTHs, and subjects with migraines. It was also observed that alterations in the perception of verticality as well as in the degrees of deviation from the perceived vertical are not related to a higher level of disability associated with neck pain; a greater frequency, intensity or disability associated with headache; or a worse quality of sleep.

Contrary to what was expected, our results showed a greater alteration of SVV in patients with migraines than in healthy controls, although without significant differences. This apparently contradictory result is in consonance with the findings of Ashish et al., 2017 [20] and Chang et al., 2019 [21] but is inconsistent with the findings of the study by Asai et al., 2009 [23]. It should be noted that, in these studies, the same measure of SVV was used as in ours (mean absolute error). The main difference between these studies is whether the head is fixed during the test. While in the study carry out by Asai et al. [23], the head was fixed at 0° during the test, in the studies conducted by Ashish et al. [20] and Chang et al. [21], the head was not fixed. Consequently, it seems that, when subject performs the test eliminating individual cervical adjustments by head fixation, the magnitude of VV deviation is greater in migraineurs than in healthy controls. However, allowing slight cervical proprioceptive adjustments during the test enables good perception of the visual vertical [20,21]. This fact suggests that, in patients with primary headache disorder, cervical afferences could act as a compensation mechanism that allows good perception of verticality. This highlights the important role played by the upper cervical structures both in the perception of verticality and in headaches. In future studies, VV measurements should be performed under different conditions and by taking into account the magnitude (absolute value) and laterality of the deviation, which would help to clarify the importance of cervical afferents and reflexes in the pathophysiology of the migraine.

Structural disorders of the upper cervical region are an important component in the pathophysiology of headaches [15,37,38,39]. In addition, headache and vestibular problems frequently occur together, giving rise to nonspecific balance disturbances concomitant to headache disorders [16,40,41]. These factors, in addition to the enormous importance of the information provided by these systems to shape the sense of verticality, are reasons why it is pertinent to look for a possible alteration in the perception of verticality in subjects complaining of headaches.

The most commonly used test to measure alteration of the visual perception of verticality is a static SVV test, which was used in this study. The most widespread interpretation is that this test mainly measures the contribution of the vestibular system to the perception of verticality [42]. In this sense, our results could be interpreted as a lack of a relationship between the alteration of the vestibular system and the appearance of headaches and migraines. Previously, other authors have evaluated SVV in similar condition to us. Although both their evaluation method and the number of measurements as well as the initial line position were different to those carried out in our study, their results are in line with our results, where no differences were found in the vestibular contribution to the perception of verticality between healthy subjects and subjects with headache disorders [20,43,44].

Another means of measuring visual verticality is the rod and frame test (RFT). In this test, a rod is displayed in darkness inside a tilted or untilted frame with respect to the earth vertical [45]. It has been suggested that the RFT measures more specifically the contribution of visual information and neck proprioception to the sensory integration of verticality [46]. Our results did not show a relationship of the vestibular contribution to the perception of verticality with headaches and migraines. Given that the RFT more specifically measures the contribution of visual and proprioceptive signals to the perception of verticality, we believe that it should be evaluated if differences are found between healthy subjects, migraineurs, and those with TTHs using the RFT as a measure of visual verticality.

Given that the internal model of space and verticality is constantly updated [47,48], it is speculated that, in these disorders, verticality alterations appear during the attack of headaches, reaching a balance during the interictal phase of the process [20]. This, together with the fact that measurements were conducted during a period in which the subjects were headache-free, could have conditioned the results obtained in our study.

There are several limitations of the present study. First, the population in which the study was carried out is very specific, which makes it difficult to extrapolate the results to populations with different characteristics. Another limitation is the difficulty of conducting these measurements at the time of the attack, restricting us to performing them during the phase in which the subjects were headache-free; this may have conditioned our results. Additionally, future studies should effectively measure the use of medication to control headache and whether this could affect verticality perception.

## 5. Conclusions

In our work, no significant differences were found in the vestibular contribution to the perception of verticality between healthy subjects or those suffering from migraines or TTHs. The variables usually related to headaches could not explain either the presence of a poor perception of verticality or the MAE during the SVV test.

For future research, it would be interesting to observe whether there are alterations in the perception of verticality during attacks as well as to observe the contribution of somatosensory and visual inputs to the perception of verticality in this and other populations. For this, it would be relevant to measure the differences in the perception of verticality between healthy and headache subjects using tests other than static SVV. 

Given the versatility of new mobile devices for measuring verticality, different tests must be implemented to be able to use them both in clinical practice and in research. This could contribute to a better understanding of the pathophysiological mechanisms that are present in patients with headaches and migraines.

## Figures and Tables

**Table 1:** Description of the samples and groups.

**Categorical Variable**		**Migraine (*n* = 28)**			**Tension-Type Headache (*n* = 74)**			**Healthy (*n* = 93)**			**Total (*n* = 195)**	
		**F**	**%**		**F**	**%**		**F**	**%**		**F**	**%**
Gender	Male	9	32.14		27	36.49		48	51.61		84	43.08
	Female	19	67.86		47	63.51		45	48.39		111	56.92
Smoker	Yes	3	10.71		11	14.86		10	10.75		24	12.31
	No	25	89.29		62	85.14		83	89.25		171	87.69
Physical activity	Yes	16	57.14		44	59.46		63	67.74		123	63.08
	No	12	42.86		30	40.54		30	32.26		72	36.92
School year	First	3	10.71		6	8.11		12	12.90		21	10.77
	Second	11	39.29		24	32.43		31	33.33		66	33.85
	Third	6	21.43		5	6.76		7	7.53		18	9.23
	Fourth	6	21.43		28	37.84		32	34.41		66	33.85
	Master	2	7.14		11	14.86		11	11.83		24	12.31
SVV	Yes	6	21.43	2.69 (0.46) *	25	33.78	2.87 (0.70) *	29	31.18	2.95 (0.66) *	60	30.77
>2.5	No	22	78.57	0.99 (0.47) *	49	66.22	0.96 (0.39) *	64	68.82	1.10 (0.50) *	135	69.23
**Continuous Variables**		**Migraine (*n* = 28)**			**Tension-Type Headache (*n* = 74)**			**Healthy (*n* = 93)**			**Total (*n* = 195)**	
		**Mean**		**SD**	**Mean**		**SD**	**Mean**		**SD**	**Mean**	**SD**
Age		20.79		2.10	21.70		4.42	21.73		3.62	21.58	3.78
Height (cm)		167.62		8.91	170.00		7.92	171.11		9.23	170.19	8.75
Weight (kg)		62.11		10.28	66.23		11.04	67.56		11.58	66.27	11.29
SVV degrees of deviation		1.36		0.84	1.61		1.04	1.68		1.02	1.61	1.01
MIDAS (Frequency of headache)		8.54		13.58	4.34		6.06	2.03		3.45	3.84	7.07
MIDAS (Intensity of headache)		5.54		1.93	5.54		2.27	2.83		2.18	4.25	2.56
MIDAS score		6.04		6.18	6.15		10.21	1.04		2.02	3.70	7.27
NDI		13.93		6.48	12.81		8.63	5.91		5.32	9.72	7.78
Sleep disturbance		41.61		15.98	40.95		13.37	36.89		15.68	39.11	14.97
Daytime somnolence		43.85		14.45	43.54		12.51	39.25		13.53	41.54	13.40
Sleep adequacy		66.37		18.49	64.19		20.07	67.38		19.34	66.03	19.46
Snoring		26.19		18.39	27.03		16.71	29.03		19.80	27.86	18.42
Awaken short		25.00		10.64	26.13		13.54	20.79		12.45	23.42	12.83
Quantity of sleep		7.43		1.23	6.91		0.89	7.09		1.16	7.07	1.08

* Mean of deviation degrees of and standard deviation for each subjective visual vertical (SVV) misperception group. MIDAS, migraine disability assessment; F, frequency; SD, standard deviation; SVV, subjective visual vertical; SF-12, 12-Item Short Form Health Survey; PCS-12, physical component summary of the SF-12; MCS-12, mental component summary of the SF-12; and NDI, neck disability index.

**Table 2:** Univariate logistic regression to analyze the factors related to alterations in the perception of verticality.

Variable	OR	95% CI		*p*-Value
		Lower	Upper	
Gender	1.087	0.533	2.219	0.818
Smoking	1.448	0.532	3.938	0.468
Physical activity	1.004	0.482	2.093	0.991
Healthy/headache	0.892	0.439	1.811	0.751
Headache Frequency	1.005	0.958	1.054	0.837
Headache intensity	1.029	0.895	1.184	0.689
MIDAS	1.023	0.981	1.067	0.280
NDI	1.014	0.970	1.060	0.535
Sleep disturbance	1.001	0.977	1.025	0.950
Daytime somnolence	1.011	0.985	1.038	0.415
Sleep adequacy	0.994	0.976	1.012	0.482
Snoring	1.011	0.993	1.029	0.225
Awaken short	1.014	0.989	1.039	0.284
Quantity of sleep	1.054	0.758	1.466	0.755

95% CI, 95% confidence interval; OR, odds ratio; MIDAS, migraine disability assessment; SF-12, 12-Item Short Form Health Survey; PCS-12, physical component summary of the SF-12; MCS-12, mental component summary of the SF-12; and NDI, neck disability index.

**Table 3:** Univariate linear regression to analyze the factors related to the degree of deviation of the perceived vertical from the theoretical vertical.

Variable	B	95% CI		*p*-Value
		Lower	Upper	
Gender	–0.026	–0.314	0.262	0.859
Smoking	0.272	–0.161	0.704	0.217
Physical activity	0.021	–0.274	0.317	0.887
Healthy/headache	–0.140	–0.425	0.145	0.334
Headache frequency	–0.005	–0.025	0.016	0.652
Headache intensity	0.003	–0.053	0.059	0.924
MIDAS	0.019	0.000	0.039	0.054
NDI	0.002	–0.017	0.020	0.868
Sleep disturbance	0.004	–0.005	0.014	0.362
Daytime somnolence	0.005	–0.006	0.015	0.405
Sleep adequacy	–0.001	–0.009	0.006	0.730
Snoring	0.004	–0.004	0.012	0.305
Awaken short	0.006	–0.006	0.017	0.323
Quantity of sleep	–0.020	–0.152	0.112	0.769

95% CI, 95% confidence interval; B, Regression coefficient; MIDAS, migraine disability assessment; F, frequency; SF-12, 12-Item Short Form Health Survey; PCS-12, physical component summary of the SF-12; MCS-12, mental component summary of the SF-12; and NDI, neck disability index.
